# The Predictive Value of Cell Blood Count Parameters to Diagnose Pulmonary Embolism in Patients with SARS-CoV-2 Infection: A Case Control Study

**DOI:** 10.3390/antibiotics11010060

**Published:** 2022-01-04

**Authors:** Alessio Strazzulla, Sarra Abroug Ben Halima, Ibrahim Chouchane, Marwa Rezek, Marcella Pinto Stiebler, Sarra Hamrouni, Mohammad Maalaoui, Nouha Ghriss, Renaud Guedec-Ghelfi, Cyrus Moini, Mehran Monchi, Nabil Belfeki

**Affiliations:** 1 Internal Medicine Unit, Groupe Hospitalier Sud Ile de France, 77000 Melun, France; sarra.abroug@ghsif.fr (S.A.B.H.); marcella.pinto-stiebler@ghsif.fr (M.P.S.); sarra.hamrouni@ghsif.fr (S.H.); mohamed.malaoui79@gmail.com (M.M.); nouha.ghriss@ghsif.fr (N.G.); nabil.belfeki@ghsif.fr (N.B.); 2 Radiology Unit, Groupe Hospitalier Sud Ile de France, 77000 Melun, France; ibrahim.chouchane@ghsif.fr; 3 Laboratory Unit, Groupe Hospitalier Sud Ile de France, 77000 Melun, France; marwa.rezek@ghsif.fr; 4 Nuclear Medicine Unit, Centre de Médecine Nucleaire, 77000 Melun, France; renaudguedec@gmail.com; 5 Cardiology Unit, Groupe Hospitalier Sud Ile de France, 77000 Melun, France; cyrus.moini@ghsif.fr; 6 Intensive Care Unit, Groupe Hospitalier Sud Ile de France, 77000 Melun, France; mehran.monchi@ghsif.fr

**Keywords:** acute pulmonary embolism, coronavirus infectious disease-2019, mean platelet volume, neutrophil-to-lymphocyte ratio, platelet-to-lymphocyte ratio, severe acute respiratory syndrome-coronavirus 2

## Abstract

*Introduction*: Acute pulmonary embolism (aPE) is frequently associated with coronavirus infectious disease-2019 (COVID-19) with an incidence of more than 16%. Among the new promising biomarkers of aPE, neutrophil-to-lymphocyte ratio (NLR) and platelet-to-lymphocyte ratio (PLR) showed correlations with aPE prognosis. The aim of this study was to conduct an exploratory analysis to check the possible role of cell blood count (CBC) parameters as diagnostic and prognostic biomarkers of aPE in COVID-19 patients. *Materials and Methods*: A case control study was conducted. Two populations were compared: (i) patients hospitalised from 31 January 2020 to 30 June 2021 with severe acute respiratory syndrome-coronavirus 2 (SARS-CoV-2) infection and aPE confirmed at angio computed tomography (aCT) or pulmonary scintigraphy (COVID-19 aPE group); (ii) patients hospitalised from 31 January 2017 to 30 June 2021 without SARS-CoV-2 infection whose suspicion of aPE was excluded by aCT or pulmonary scintigraphy (no-aPE group). *Results*: Overall, 184 patients were included in the study, 83 in COVID-19 aPE group and 101 in no-aPE group. At the univariate analysis, COVID-19 patients with aPE had higher NLR, PLR, neutrophil and lymphocyte counts than patients without aPE (*p* < 0.05). No significant difference was found in mean platelet volume and platelet counts. No difference in mortality rate was detected. At the multivariate analysis, neutrophil and lymphocyte counts were both associated with diagnostic of aPE while no CBC parameters were associated with mortality at day#7. *Conclusions*: Neutrophiland lymphocyte counts could be predictors of the early detection of aPE in COVID-19 patients. The value of CBC indices as biomarkers of aPE in daily clinical practice needs to be investigated in further studies.

## 1. Introduction

Since its outbreak in late 2019 the coronavirus infectious disease-2019 (COVID-19) pandemic had caused 260,867,011 confirmed cases and 5,200,267 deaths by 29 November 2021 [1]. Among COVID-19 complications, acute pulmonary embolism (aPE) is a cause of morbidity and mortality and it is observed in 16% of COVID-19 patients [2]. Diagnosis of aPE is based on angio-computed tomography (aCT) and nuclear medicine exams, such as lung scintigraphy [3,4]. However, their systematic use in COVID-19 patients is limited by their costs, their unavailability in some medical facilities and hospital overcrowding, especially during COVID-19 epidemic rebounds. Consequently, the suspicion of aPE and the realization of radiology and nuclear medicine exams need to be guided by other diagnostic tools, the most common of which are Wells score and D-Dimer dosage. Unfortunately, both these tools are affected by low sensitivity or specificity in case of COVID-19. Indeed, Wells score in COVID-19 patients is reliable only when it is equal or superior to 4 while D-Dimer assay needs a higher threshold than not-COVID patients to achieve a satisfactory specificity [5,6]. Thus, other predictive markers are needed for address suspicion of aPE in COVID-19 patients and reduce the risk of short-term events.

Cell blood count (CBC) was proposed as prognostic tool of aPE. Among CBC parameters, neutrophil-to-lymphocyte ratio (NLR) and platelet-to-lymphocyte ratio (PLR) are promising prognostic factors of aPE. As a matter of fact, they were both associated with short and long term mortality in aPE [7]. Moreover, some promising results showed that mean platelet volume (MPV) correlated with diagnosis of deep venous thrombosis (DVT) and prognosis of aPE [8,9]. On the other hand, the role of CBC parameters for the prediction of aPE diagnosis and evolution in patients affected by severe acute respiratory syndrome-coronavirus 2 (SARS-CoV-2) infection is still unclear [10]. Indeed, many abnormalities in coagulation parameters were signalled, such as increase of fibrin degradation products, thrombopenia, prolongation of protrombin and activated partial thromboplastin time [11]. NLR seemed to be a marker of inflammation and it was associated with hospital readmission in previously discharged COVID-19 patients [12,13].

The aim of the study was to conduct an exploratory analysis to verify the possible role of CBC parameters as diagnostic and prognostic biomarkers of aPE in COVID-19 patients.

## 2. Materials and Methods

A case control study was conducted in a single centre in the *Ile de France* region in France. Patients included in the study were hospitalised in a general hospital from 31 January 2017 to 30 June 2021. Two populations were compared: (i) patients hospitalised from 31 January 2017 to 30 June 2021 without SARS-CoV-2 infection whose suspicion of aPE was excluded by aCT or pulmonary scintigraphy (no-aPE group); (ii) patients hospitalised from 31 January 2020 to 30 June 2021 with SARS-CoV-2 infection and aPE confirmed at aCT or pulmonary scintigraphy (COVID-19 aPE group). All patients with SARS-Cov-2 infection were tested positive to SARS-Cov-2 polymerase chain reaction (PCR) in nasopharyngeal swab. Exclusion criteria included: (i) age <18 years; (ii) absence of aCT or scintigraphy; (iii) consecutive hospitalisations.

The study was conducted in accordance with the Declaration of Helsinki and national and institutional standards. According to the French law, consent obligation and patient’s written consent were not waved by the local institutional review board because of the retrospective character of the study [14,15].

Data collection was obtained through software used in daily clinical practice (Sillage v17 and CGM Lab channel 1.20.33686). The following data were collected at hospital admission: age, gender, aPE risk factors (cancer, haemopathy, acquired thrombophilia, stroke, heart failure sepsis, recent surgery, immobilization), cell blood count, vital parameters (oxygen saturation, respiratory rate, systolic blood pressure, heart rate, pulmonary embolism severity index (PESI) score.

The main clinical outcome was mortality at seven days from aPE diagnosis (mortality at day#7). Secondary outcomes were: (i) mortality at one month from aPE diagnosis (mortality at month#1); (ii) mortality at three months from aPE diagnosis (mortality at month#3).

Chi-squared test (qualitative variables) and Student’s T-test (quantitative variables) were applied for the univariate analysis. Differences in clinical outcomes and clinical characteristics of patients included in the two groups were compared (COVID-19 aPE group vs. no-aPE group). Quantitative variables were presented in the text as mean values. A multiple logistic regression analysis was performed to explore CBC parameters correlated with aPE diagnosis and mortality at day#7. CBC parameters included in multivariate analysis were chosen according to univariate analysis results (*p* ≤ 0.050). Analyses were performed using R, the Language for Statistical Computing (Vienna, Austria, https://www.r-project.org/ accessed on 26 October 2021). Statistical significance was set at *p* < 0.050.

## 3. Results

Overall, 184 patients were included in the study, 83/184 (45%) in COVID-19 aPE group and 101/184 (55%) in no-aPE group.

At the univariate analysis, no difference in mean age and gender distribution was found. Patients in COVID-19 aPE group had higher NLR (*p* = 0.0015,) PLR (*p* = 0.0085), neutrophil (*p* = 0.0079) and lymphocyte counts (*p* = 0.0039), haemoglobin (*p* = 0.0002), and packed cell volume or PCV (*p* = 0.0002) than patients without aPE. Red cell distribution width (RDW) was lower in COVID-19 aPE group than no-aPE group (*p* = 0.0073). No significant difference was found in mean platelet volume and platelet counts. Among clinical outcomes, no difference in mortality rate was detected while length stay was higher (*p* = 0.0006) in COVID-19 aPE group than no-aPE group (Table 1).

At the multivariate analysis, neutrophil and lymphocyte counts were both independently associated with diagnostic of aPE (Table 2) while no CBC parameters were associated with mortality at day#7 (Table 3).

## 4. Discussion

This article showed for the first time the potential role of neutrophil and lymphocyte counts as diagnostic biomarkers of aPE in COVID-19 patients. The role of NLR and PLR remains not entirely defined whereas platelet counts and MPV do not seem to have any correlation with aPE in COVID-19 patients according to results of this study.

The primary finding of this study was the association of neutrophil and lymphocyte counts with diagnosis of aPE in patients with SARS-CoV-2 infection. The association of high neutrophil counts and low lymphocyte count with severe COVID-19 is well known since early days of pandemic in China but no study had found a specific association between neutrophil and lymphocyte count and COVID-19 complications [16]. However, neutrophil and lymphocyte counts are markers of acute phase and they can be influenced by numerous factors not explored in this study. Because of this high variability, the use of neutrophil and leukocyte counts as aPE marker in COVID-19 patients should be taken with extreme caution.

The most diffused biomarker of aPE is D-Dimer. However, D-Dimer is constantly increased in COVID-19 patients and cut-offs need to be increased to achieve a satisfactory specificity with, conversely, a significant reduction of sensibility for the prediction of aPE [2]. Consequently, its routinely dosage in COVID-19 patients cannot be recommended. At the same time, Wells score underestimates whereas Geneva score overestimates the risk of aPE in COVID-19 patients [5]. As a matter of fact, probability scores include only clinical parameters without considering the key role of inflammation to explain the increased risk of aPE in COVID-19 patients. In fact, a cytokine storm is observed in COVID-19, including pro-inflammatory cytokines involved in abnormal clot formation, platelet hyper-activation and anticoagulant pathway regulation [17]. Although many studies tried to describe the role of pro-inflammatory cells in COVID-19 pathogenesis, no cytokine was specifically associated with aPE pathogenesis, thus a dosage as marker of aPE cannot be proposed for any cytokynes [18]. This study gave for the first time evidence of a possible utility of neutrophil and lymphocyte count dosage as biomarkers of aPE in COVID-19 patients.

This study failed in demonstrating that NLR and PLR were independently associated with diagnosis of aPE and mortality. This result is partially deceiving and it contrasts with literature. In fact, different studies reported a relevant impact of NLR and PLR on short term mortality in patients affected by aPE [7,19]. We can argue that our study was performed in a different and never explored context. Indeed, to our best knowledge, we evaluated the role of NLR and PLR as biomarker of aPE in COVID-19 patients for the first time. Thusthe presence of SARS-Cov-2 could have biased the overall mortality rate. Unfortunately, the study design does not make it possible to distinguish between aPE related and COVID-19 related deaths. Notwithstanding these deceiving results, we think that the use of NLR and PLR in COVID-19 as marker of inflammation should be further investigated, as suggested by results of former studies [12,13].

The role of platelet indices (platelet counts and MPV) as marker of severity of aPE is still controversial. The increase in platelet size is associated with increased adhesiveness and this association seems to find its clinical confirmation by some studies which showed a correlation between elevated MPV and severity of aPE [9,20,21]. However, other studies showed that a single MPV dosage at aPE diagnosis did not correlate with aPE severity [22]. Rather, MPV progressive increase at end of aPE treatment correlated with severity of aPE and with the risk of aPE recurrence [23,24]. We contribute to debate with our results showing no significant modification of baseline platelet counts and MPV with the diagnosis of aPE and mortality in COVID-19 patients.

We conducted a monocentric retrospective study. As a consequence, our results are not automatically applicable todifferent realities. Besides, the number of patients included in the study was limited and a partial loss of data is inevitable because of the study design. On the whole, the results of this study need to be confirmed by further studies. A comparisonof CBC parameters in COVID-19 patients with or without aPE needs to be performed. A follow-up of CBC parameters before, during and after the diagnosis of aPE is also required. Finally, a larger multicentric study is needed to validate or not our findings.

## 5. Conclusions

For the first time, this study showed the potential role of neutrophil and lymphocyte count as biomarker of aPE in COVID-19 patients. NLR, PLR, platelet counts and MPV did not demonstrate having a correlation with aPE in COVID-19 patients. The value of CBC indices as biomarkers of aPE in daily clinical practice needs to be investigated in further studies.

## Figures and Tables

**Table 1:** Characteristics of Patients.

Caracteristics	COVID-19 aPE		No-aPE		*p*-Value
	n = 83		n = 101		
**Biological Parameters**					
Age (years), mean [SD]	57	[16.7]	59	[18.3]	0.3913
Male Gender, n [%]	49	[59]	54	[53]	0.0590
**RiskFactors**					
Cancer, n [%]	4	[4.8]	15	[14.9]	0.0394
Haemopathy, n [%]	1	[1.2]	3	[3.0]	0.7300
Acquired Thrombophilia, n [%]	0	[0]	1	[0.9]	0.3657
Stroke, n [%]	0	[0]	0	0	1
Heart Failure, n [%]	6	[2.7]	8	[7.9]	0.9025
Sepsis, n [%]	5	[0.6]	11	[10.9]	0.3657
Recent Surgery, n [%]	1	[1.2]	4	[3.9]	0.2645
Immobilization, n [%]	4	[4.8]	11	[9.10]	0.1074
**Cell Blood Count**					
Leukocytes (G/L), mean [SD]	10.2	[3.8]	8.3	[4.0]	0.0896
Neutrophils (G/L), mean [SD]	7.1	[3.8]	5.6	[3.9]	0.0079
Lymphocytes (G/L), mean [SD]	1.2	[0.6]	1.7	[0.8]	0.0039
Haemoglobin (g/dL), mean [SD]	13.2	[0.6]	11.8	[2.6]	0.0002
PCV (%), mean [SD]	39.9	[5.5]	35.8	[3.7]	0.0002
RDW (%), mean [SD]	13.5	[1.7]	14.4	[2.0]	0.0073
Platelets (G/L), mean [SD]	272	[108]	250	[101]	0.1693
MPV (fl), mean [SD]	10.3	[1.1]	10.4	[1.4]	0.1936
Neutrophil-to-Lymphocyte Ratio, mean [SD]	7,5	[6.6]	3.2	[1.8]	0.0015
Platelet-to-lymphocyte Ratio, mean [SD]	259	[159]	204	[153]	0.0085
**Vital Parameters**					
Oxygen Saturation (%), mean [SD]	88	[16]	95	[14]	0.0315
Respiratory Rate (acts/minute), mean [SD]	26	[9]	20	[6]	0.0003
Systolic Blood Pressure (mmHg), mean [SD]	135	[34]	133	[28]	0.6206
Heart Rate (beats)%, mean [SD]	94	[18]	88	[18]	0.0388
Score PESI, mean [SD]	89	[33]	76	[32]	0.0513
**Outcomes**					
Mortality at Day#7, n [%]	3	[3.6]	1	[0.9]	0.2134
Mortality at Month#1, n [%]	6	[7.2]	3	[2.9]	0.2987
Mortality at Month#3, n [%]	6	[7.2]	5	[4.9]	0.7121
Length of stay (days), mean [SD]	10	[10.3]	3	[2.9]	0.0006

Abbreviations: COVID-19 = coronavirus infectious diseases-2019; MPV = mean platelet volume; PCV = packed cell volume; aPE = acute pulmonary embolism; RDW = red cell distribution width.

**Table 2:** Logistic Regression Analysis (Acute Pulmonary Embolism Diagnosis).

Parameters	OR (95%CI)	*p*-Value
Neutrophils	1.20 (1.04–1.40)	0.0153
Lymphocytes	0.45 (0.23–0.86)	0.0168
Haemoglobin	0.89 (0.51–1.55)	0.6900
PCV	1.15 (0.94–1.40)	0.1668
RDW	0.76 (0.58–1.00)	0.0503
Neutrophil-to-lymphocyte ratio	0.93 (0.85–1.02)	0.1285
Platelet-to-lymphocyte ratio	1.00 (0.99–1.00)	0.3018

Abbreviations: PCV = Packed Cell Volume; RDW = Red Cell Distribution Width.

**Table 3:** Logistic regression analysis (Mortality at day#7).

Parameters	OR (95%CI)	*p*-Value
Neutrophils	1.18 (0.82–1.70)	0.3702
Lymphocytes	1.17 (0.43–3.19)	0.7571
Haemoglobin	1.02 (0.44–2.38)	0.9641
PCV	0.96 (0.71–1.30)	0.8109
RDW	1.12 (0.77–1.64)	0.5452
Neutrophil-to-Lymphocyte Ratio	1.02 (0.75–1.40)	0.8866
Platelet-to-Lymphocyte Ratio	1.00 (0.99–1.00)	0.6000

Abbreviations: PCV = Packed Cell Volume; RDW = Red Cell Distribution Width.

## Data Availability

Data were available from correspondent author after motivated question.

## Abbreviations

aCT: angioComputed Tomography; aPE: acute Pulmonary Embolism; CBC: Cell Blood Count; COVID-19: Coronavirus Infectious Disease-2019; DVT: Deep Venous Thrombosis; MPV: Mean Platelet Volume; NLR: Neutrophil-to-Lymphocyte Ratio; PCR: Polymerase Chain Reaction; PCV: Packed Cell Volume; PESI: Pulmonary Embolism Severity Index; PLR: Platelet-to-Lymphocyte Ratio; RDW: Red Cell Distribution Width; SARS-CoV-2: Severe Acute Respiratory Syndrome-Coronavirus 2.
